# On the Respiration of Cold Air in Pulmonary Diseases

**Published:** 1829-04-01

**Authors:** 


XLIX.

### On the Respiration of Cold Air in Pulmonary Diseases. By Dr. Drake, of New York.

Dr. Drake, some time ago, published a letter on this subject, which we noticed in a former number of this Journal. He has since been extending the trial of respiration of cool air to a considerable number of additional cases, with beneficial, though not uniformly beneficial effects. The greater number of cases were long standing chronic catarrhs, some of which had been preceded by hæmoptysis—some presented indications of slight irregular hectic, with more or less expectoration — "and in one, pectoriloquy was distinctly heard in the right lung." None of these, however, exhibited symptoms of much inflammatory excitement, beyond a certain frequency of the pulse, and soreness of the chest.

"As to the temperature of the air for respiration, I have found that it may be reduced very low with advantage. In some instances I have reduced it to zero, and even a few degrees below, which patients have preferred to a higher degree; but in general, I have contented myself with employing ice simply, which brought the air to 32° or 34°, and this seemed to answer very well.

"As the warm weather has advanced, I have been less attentive than formerly in maintaining external stimulation by means of the vest or irritating plasters, and I do not find the remedy less efficacious on that account, or the neglect attended by any ill effect, except perhaps that such patients, after respiring very cold air for a considerable time, are more liable to complain of general coldness, amounting almost to a rigor, than when the chest has been kept warmly clad or stimulated. In no instance, however, did any ill consequence arise from this state of things, which I attribute to the blood being diverted from, instead of being accumulated in the diseased organ, as happens when a chill arises from exposure of the external surface to cold and moisture.

"I have made a number of experiments, both in pulmonic cases and on healthy individuals, for the purpose of ascertaining the effects of cool respiration on the pulse. In the great majority of instances the pulse was rendered both slower and fuller; sometimes only fuller, and now and then somewhat more frequent. One or two hours respiration of air brought to zero, or even 10° above, would usually reduce the pulse from 16 to 24 beats, provided it was not much above a hundred; but I was not successful in making a decided impression on it when it was beyond that point in frequency. Forty-seven was the slowest I was enabled to render it, when it rose up under the finger very full and sluggishly. In one healthy individual it was reduced in two hours from 88 to 52, in another from 72 to 56, and in a third from 80 to 64 in one hour.

"After all, it will require a much more extended experience than I have yet had, especially during the heats of summer, to form a correct estimate of the value of this remedy, and to ascertain the particular forms of pulmonic disease in which it may be advantageously employed, for doubtless it will not be found suited to all indiscriminately."*

A section is given of an apparatus for the exhibition of cool air—but such an apparatus may be very easily devised by any one who wishes to try the remedy. It is not a little curious to find cold air recommended for that very class of complaints in which a warm and regulated temperature of the atmosphere is considered so beneficial. The very circumstance, however, of pulmonary diseases bearing, with impunity, this local refrigeration, may suggest the propriety, in some cases at least, of gradual exposure to the pure and open air, where, according to present notions, the patient should be kept confined in closed apartments, under artificial heat. We fear that *this* system is carried too far, and that the constitution is often enervated *by* this cookery, without any real check being given to the progress of disease in the lungs.

* American Journal of the Med. Sciences, No. V.

